# Curcumin supplementation could improve diabetes-induced endothelial dysfunction associated with decreased vascular superoxide production and PKC inhibition

**DOI:** 10.1186/1472-6882-10-57

**Published:** 2010-10-14

**Authors:** Sirada Rungseesantivanon, Naris Thenchaisri, Preecha Ruangvejvorachai, Suthiluk Patumraj

**Affiliations:** 1 Inter-department of Physiology, Graduate School, Chulalongkorn University, Bangkok 10330, Thailand; 2 Department of Companion Animal Clinical Sciences, Faculty of Veterinary Medicine, Kasetsart University, Bangkok 10900, Thailand; 3 Department of Pathology, Faculty of Medicine, Chulalongkorn University, Bangkok 10330, Thailand; 4 Department of Physiology, Faculty of Medicine, Chulalongkorn University, Bangkok 10330, Thailand

## Abstract

**Background:**

Curcumin, an Asian spice and food-coloring agent, is known for its anti-oxidant properties. We propose that curcumin can improve diabetes-induced endothelial dysfunction through superoxide reduction.

**Methods:**

Diabetes (DM) was induced in rats by streptozotocin (STZ). Daily curcumin oral feeding was started six weeks after the STZ injection. Twelve weeks after STZ injection, mesenteric arteriolar responses were recorded in real time using intravital fluorescence videomicroscopy. Superoxide and vascular protein kinase C (PKC-βII) were examined by hydroethidine and immunofluorescence, respectively.

**Results:**

The dilatory response to acetylcholine (ACh) significantly decreased in DM arterioles as compared to control arterioles. There was no difference among groups when sodium nitroprusside (SNP) was used. ACh responses were significantly improved by both low and high doses (30 and 300 mg/kg, respectively) of curcumin supplementation. An oxygen radical-sensitive fluorescent probe, hydroethidine, was used to detect intracellular superoxide anion (O_2_^●-^) production. O_2_^●- ^production was markedly increased in DM arterioles, but it was significantly reduced by supplementation of either low or high doses of curcumin. In addition, with a high dose of curcumin, diabetes-induced vascular PKC-βII expression was diminished.

**Conclusion:**

Therefore, it is suggested that curcumin supplementation could improve diabetes-induced endothelial dysfunction significantly in relation to its potential to decrease superoxide production and PKC inhibition.

## Background

Diabetes mellitus (DM) is characterized by chronic hyperglycemia and its developed diabetic complications, in particular, macroangiopathy and microangiopathy. These pathophysiological complications are often responsible for a decreased quality of life in diabetic patients [1]. Experimental evidence indicates that hyperglycemia induces a series of cellular events that increase the production of reactive oxygen species (ROS) [2]. In the vessel, one of the most important ROS is superoxide anion (O2●-), which is formed by the univalent reduction of oxygen [3]. There are multiple enzymes involved in the production of O2●- and its derivatives in the vasculature, in particular, vascular protein kinase C (PKC)-activated NAD(P)H oxidase [4]. The O2●- produced can inactivate nitric oxide (NO) [5,6] directly, which leads to decreased NO bioavailability [7,8]. NO is an important molecule that involves many vascular functions. The diabetes-induced increase in O2●- and its relation to diabetic vascular complications have attracted a lot of attention from several investigators. In animal models of diabetes, antioxidant defense capacities were diminished in certain tissues [9]. In addition, human and animal studies have attempted to restore vascular endothelial function using different types of antioxidants [10-12]. However, a critical evaluation of clinical trials suggests a difference in the ROS specific to various vascular diseases, thereby limiting the effectiveness of specific antioxidants [13]. Various herbal extracts are known to possess antioxidant properties. Curcumin, a yellow pigment from the root of *Curcuma longa *Linn., is a major component of turmeric and commonly used as a spice and food-coloring agent. Anti-oxidant and anti-inflammatory properties of curcumin have been well documented by previous studies [14-16]; however, the effect of curcumin, especially on diabetes-induced vascular O_2_^●- ^production, remains to be clarified. Recently, it has been reported that curcumin (300 mg/kg) could enhance the effect of vitamin C in protecting endothelial cells, through an anti-oxidant effect [17]. Therefore, in the present study, we tested the effects of curcumin supplementation by using two different doses of 30 mg/kg and 300 mg/kg on diabetes-induced endothelial dysfunction, which is associated with the direct effects on vascular O_2_^●- ^production. We also examine the potential of curcumin in inhibiting diabetes-induced PKC-activation by using immunofluorescent staining.

## Methods

Male Wistar rats were housed in a temperature- and light-controlled environment, fed standard chow and had acess to tap water *ad libitum*. The present study was conducted in accordance with the guidelines for animal experimentation established by the National Research Council of Thailand and approved by the Institutional Animal Care and Use Committee of Chulalongkorn University.

### Induction of diabetes

The rats were randomly divided into non-diabetic and diabetic groups. Diabetes was induced by a single intravenous injection of streptozotocin (55 mg/kg, STZ, Sigma-Aldrich Co., USA). STZ was freshly prepared by dissolving it in citrate buffer (pH 4.5, Sigma-Aldrich Co., USA) and immediately injected into the tail vein after 8 hours of fasting. Control rats received citrate buffer of the same volume instead. STZ-induced diabetic rats were included and retained for the experiments if their blood glucose was greater than 200 mg/dL. Blood glucose was measured by using a glucometer (ACCU-CHEK, ADVANTAGE, Roche Diagnostics, Germany). Animals were separated in five groups: (1) diabetes (DM; n = 10), (2) DM-treated with curcumin (Cayman Chemical Co., USA) 30 mg/kg (DM+cur30; n = 10), (3) DM-treated with curcumin 300 mg/kg (DM+cur300; n = 10), (4) control (con; n = 10), and (5) control treated with 300 mg/kg (con+cur300; n = 10). It is noted that the daily oral feeding of curcumin was started at six weeks after the STZ injection, since it has been shown by our previous study that endothelial dysfunction in STZ-rats has already occurred at six-weeks after STZ injection [11].

### Intravital observation of mesenteric arteriolar responses

Twelve weeks after STZ injection (or vehicle), the rats were anesthetized with an intraperitoneal injection of pentobarbital sodium (50 mg/kg). After tracheostomy, polyethylene tubes were inserted into the external jugular vein and the common carotid artery for injection of fluorescence tracers and monitoring of blood pressure, respectively. The abdominal cavity was opened via midline incision. The rat was placed on its right side on a microscope stage. A small loop of intestine was exteriorized. The mesentery was carefully spread on a plexiglass chamber with continuous perfusion by 1 mL/min Krebs-Ringer buffer (37°C, pH 7.4, bubbled with 95% O_2 _and 5% CO_2_, composition in mmol/L: 135.7 NaCl, 4.7 KCl, 2.52 CaCl_2_, 1.18 KH_2_PO_4_, 1.64 MgSO_4_.7H_2_O, and 7.14 NaHCO_3_).

The second-order mesenteric arterioles (20 to 35 μm in diameter) were viewed and recorded in real time by an epi-illumination fluorescence videomicroscopy system (Optiphot 2, Nikon, Japan) equipped with a 100 W mercury lamp, CCD camera (Hamamatsu C2400, Japan), a video recorder (VC-S5, Sharp, Japan) with a video timer (VTG-33, For-A, Japan) and a 20× objective lens (CF Plan Fluor, Nikon, Japan). Un-branched segments of mesenteric arterioles were visualized by fluorescein isothiocyanate-labeled dextran (FITC-Dextran 250, 5 μg/mL, Sigma-Aldrich Co., USA). The mesenteric arterioles were allowed 15 minutes of stabilization and pre-constricted with norepinephrine (10^-5 ^mol/L, NE). Topical applications of two vasodilators, acetylcholine (10^-5 ^mol/L, ACh) and NO donor sodium nitroprusside (10^-5 ^mol/L, SNP) were used to test the functions of the endothelium and smooth muscle of mesenteric arterioles, respectively. Changes in mesenteric arteriolar diameters were analyzed off-line using digital image software (Image-Pro Plus; Media Cybernetics, Inc., USA) and expressed as the percentage of relaxation after pre-constriction with NE by using the equation: [(D_ACh_-D_NE_)/D_NE _× 100].

### Direct detection of superoxide content using ethidium bromide fluorescence

**Figure 1:**
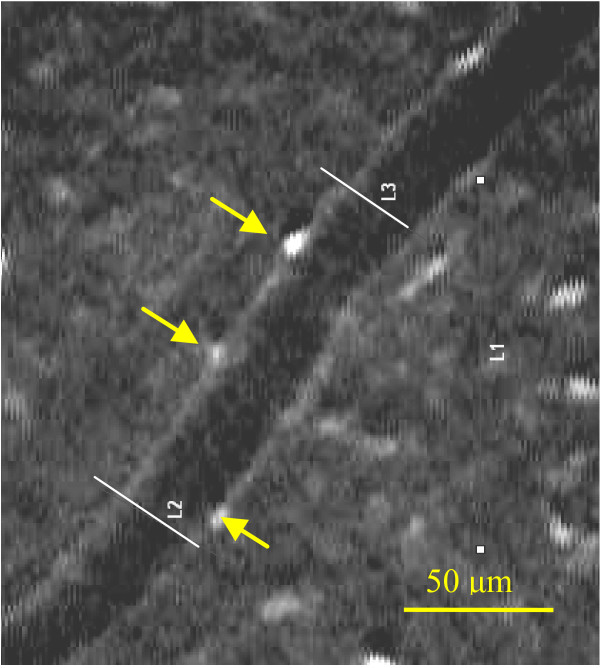
**Ethidium bromide-positive nuclei**. Number of ethidium bromide (EB)-positive nuclei from the selected arteriolar wall. The white lines depict the 100-micron vascular length where EB-positive nuclei were counted. (Bar represents 50 μm).

Mesenteric arterioles were subjected to O_2_^●- ^measurement based on fluorescence detection using the DNA-binding fluorophore ethidium bromide (EB), which is formed by O_2_^●- ^oxidation of hydroethidine (HE), using 490 nm excitation and 590 nm emission wavelengths [18]. The mesentery preparation was perfused with a buffer solution containing hydroethidine (5.0 × 10^-6 ^mol/L, Polysciences, USA) saturated with a 95% N_2 _and 5% CO_2 _gas mixture for 60 minutes according to the modified method described by Suzuki *et al*. [19,20]. The number of nuclei labeled with ethidium bromide (EB-positive nuclei) along the mesenteric arteriolar wall was determined at 60 minutes after the onset of hydroethidine perfusion. The number of EB-stained nuclei was counted per 100 microns of vessel length, as shown in Figure 1. The selection of microvessels was limited to arterioles with diameters ranging between 20 to 35 μm. The results were confirmed by the other examiner, who was blinded to grouping while counting.

### Immunofluorescent staining for PKC-βII

Single unbranched small mesenteric arteries with diameter ~100 μm were selected for study. The selected microvessels were post-fixed in 4% paraformaldehyde for 24 hours and were embedded in paraffin. These specimens were then deparaffinized in xylene, rehydrated in graded ethanol and distilled water, antigen unmasked with sodium citrate (10 mmol/L, pH 6.0, Dako, Denmark), and then exposed to a microwave heat source. Incubation with anti-PKC-βII (1:100 dilution, Santa Cruz Biotechnology, CA) was performed at room temperature for 60 minutes. Sections were then washed in PBS and incubated with the secondary antibody swine anti-rabbit IgG-TRITC (1:50 dilution, Dako, Denmark) for 30 minutes at room temperature. Labeling of the arteries with secondary antibody alone was used as negative control. Images were obtained using laser scanning confocal microscopy (E800, Nikon, Japan) to establish the localization of PKC-βII in small mesenteric arteries.

### Measurement of plasma glucose and HbA1c

At the end of each experiment, a blood sample was collected from each rat for further plasma glucose and glycosylated hemoglobin (HbA1c) determination, using the enzymatic and turbidimetric immunoinhibition methods, respectively (Bangkok RIA Laboratory Co., Ltd., Bangkok, Thailand).

### Statistical analysis

Data were expressed as means and standard errors of means (SEM). For comparison among groups, one-way analysis of variance (one-way ANOVA) and Tukey post-hoc test were used. *P *< 0.01 and < 0.05 were considered statistically significant. All data were analyzed using the SPSS program (version 16.0) for Windows.

## Results

### Effects of curcumin on mean arterial blood pressure, plasma glucose, and HbA1c levels

Twelve weeks after the injection of STZ, plasma glucose and HbA1c values were significantly elevated in DM rats as compared with control rats (Table 1). Supplementation of curcumin for six weeks in control rats did not alter the plasma glucose level. Interestingly, the high-dose supplementation of curcumin in the DM+cur300 group significantly lowered the levels of both plasma glucose and HbA1c in comparison to the DM group (*P *< 0.05). The low dose of curcumin supplementation (30 mg/kg) resulted in a slight decrease in plasma glucose and significantly lower levels of HbA1c when compared to values observed in the DM group (DM = 10.73 ± 0.32, DM+cur30 = 8.20 ± 0.88) (*P *< 0.05). However, treatment with a high dose of curcumin (300 mg/kg) resulted in only a 32% decrease in plasma glucose (DM = 459.0 ± 24.40, DM+cur300 = 310.00 ± 32.73), and the values of plasma glucose and HbA1c of both DM+cur30 and DM+cur300 were significantly increased when compared to the controls (*P < 0.01*). Therefore, it is noted that the hyperglycemic state still existed in both groups, DM+cur30 and DM+cur300.

**Table 1:** Mean arterial blood pressure (mABP; mmHg), plasma glucose (mg/dl), and glycosylated hemoglobin (HbA1c, %)

Group	mABP (mmHg)	Plasma glucose (mg/dL)	HbA1c (%)
control	103.1 ± 3.57	101.8 ± 4.89	3.68 ± 0.17
con+cur300	106.18 ± 3.93^NS^	106.8 ± 0.92^NS^	4.08 ± 0.41^NS^
DM	151.67 ± 9.68**	459.0 ± 24.40**	10.73 ± 0.32**
DM+cur30	128.33 ± 3.57	360.8 ± 35.82**	8.20 ± 0.88**, †
DM+cur300	122.27 ± 8.68†	310.00 ± 32.73**, †	7.90 ± 0.97**, †

Values are means ± SEM (n = 10) from the groups of: 12-week STZ (DM), diabetes treated with curcumin (DM+cur30, DM+cur300), controls (con) and controls treated with curcumin (con+cur300). NS, no significant difference compared to control, **P < 0.01, significant difference compared to control and control treated with curcumin; † *P *< 0.05, significant difference from diabetic rats.

In comparison with the control group, mean arterial blood pressure (mABP) was significantly increased in the DM group (*P *< 0.01). This increased mABP was significantly attenuated with a high dose of curcumin supplementation (*P *< 0.05).

### Effects of curcumin on mesenteric arteriolar responses

**Figure 2:**
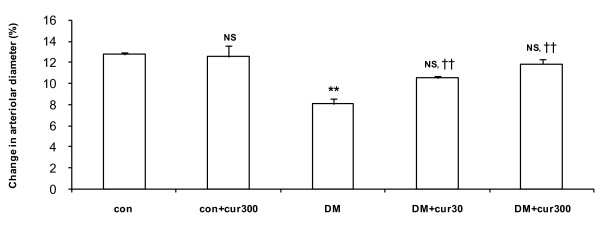
**Acetylcholine-induced arteriolar vasodilation**. Acetylcholine-induced changes in mesenteric arteriolar diameters from control (con), diabetes (DM) and curcumin-treated groups (DM+cur30, DM+cur300 and con+cur300). Data are means ± SEM (n = 5 for each group). NS, not significant different compared to control arterioles; ** *P *< 0.01, significant difference compared to control arterioles; †† *P *< 0.01, significant difference compared to diabetic arterioles.

The dilatory response of the mesenteric arterioles to ACh (10^-5 ^mol/L) was significantly decreased in the diabetic group (8.11 ± 0.44%) as compared to the control group (12.82 ± 0.2%, *P *< 0.01) (figure 2). Both low and high doses of curcumin supplementation (DM+cur30; 10.56 ± 0.2% and DM+cur300; 11.88 ± 0.52%) significantly restored arteriolar dilation in response to ACh (10^-5 ^mol/L) in comparison to the physiology observed in DM rats (8.11 ± 0.44%, *P *< 0.01). However, supplementation with curcumin in the con+cur300 group did not show any effects on ACh-induced arteriolar dilation as compared to control (12.58 ± 1.07% and 12.82 ± 0.2%, respectively).

**Figure 3:**
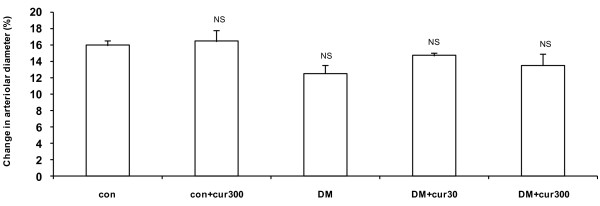
**Sodium nitroprusside-induced arteriolar vasodilation**. Sodium nitroprusside-induced changes in mesenteric arteriolar diameter from control (con), diabetes (DM) and curcumin-treated groups (con+cur300, DM+cur30, DM+cur300). Data are means ± SEM (n = 5 for each group). NS, no significant difference compared to control arterioles.

Figure 3 demonstrated that the impaired vasodilatation in mesenteric arterioles of DM rats appeared to involve only endothelial cell function but not smooth muscle function because the vasodilation response to SNP-activation was not altered in DM (12.55 ± 0.95%), DM+cur30 (14.71 ± 0.38%), or DM+cur300 groups (13.54 ± 1.40%).

### Effect of curcumin on vascular superoxide production

**Figure 4:**
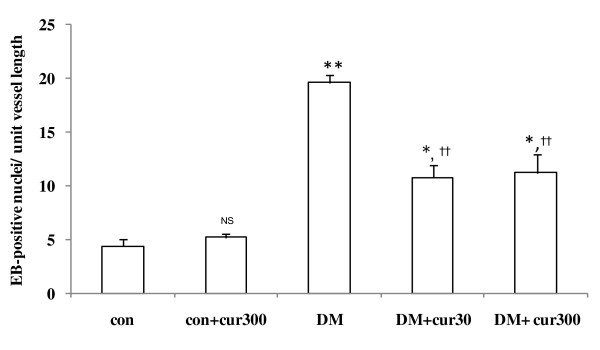
**Number of ethidium bromide-positive nuclei**. Histogram showing the ethidium bromide-positive nuclei along the mesenteric arterioles of rats that were untreated diabetics (DM), diabetics treated with low curcumin (DM+cur30), diabetics treated with high curcumin (DM+cur300), controls (con) or controls treated with curcumin (con+cur300) rats. Data are expressed as mean ± SEM (n = 5 for each group). NS, no significant difference compared to control arterioles; **P < 0.01 and *P < 0.05, significant difference compared to control arterioles; †† *P *< 0.01, significant difference compared to diabetic arterioles.

By using hydroethidine-sensitive vascular superoxide, the results showed that the number of EB-positive nuclei per 100-micron vessel length were significantly increased along the vascular walls of DM rats (19.6 ± 0.8) as compared to control rats (4.4 ± 0.6, *P *< 0.01). The numbers of EB-positive nuclei observed in DM+cur30 and DM+cur300 groups (10.8 ± 1.2 and 11.2 ± 1.8, respectively) were significantly reduced as compared to DM (19.6 ± 1.8, *P *< 0.01) but remained higher than levels observed in controls (4.4 ± 0.6, *P *< 0.05) (figure 4). There was no significant difference between the EB-positive nuclei in control and con+cur300 arterioles (4.4 ± 0.6 and 5.2 ± 0.4 respectively).

### Effect of curcumin on PKC-βII in mesenteric artery

**Figure 5:**
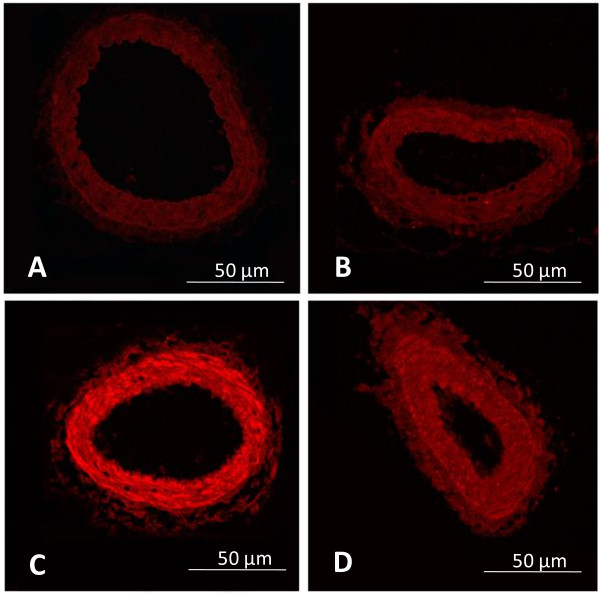
**Immunofluorescent staining for PKC-βII**. PKC-βII and TRITC signals from immunofluorescent staining of mesenteric arteries. Microvessels with diameter of approximately 100 μm were fixed in 4% paraformaldehyde for 24 hours and then embedded in paraffin. They were later deparaffinized in xylene and rehydrated in a mixture of ethanol and distilled water. Antigens were unmasked using sodium citrate (10 mmol/L, pH 6.0), followed by exposure to a microwave heat source. Samples were then incubated at room temperature for 60 minutes with anti-PKC-βII at 1:100. Sections were washed in PBS and incubated with swine anti-rabbit IgG-TRITC (1:50 in PBS) for 30 minutes at room temperature. Immunofluorescent staining of small mesenteric arteries displayed a strong signal for PKC-βII in DM rats (Figure 5C). In contrast, the TRITC signals of anti-PKC-βII antibodies were weak in the controls and DM+cur rat vessels (Figure 5 B and D, respectively). The negative control displayed a minimal detectable fluorescence when the secondary antibodies were used alone (Figure 5A).

Immunofluorescent staining of small mesenteric arteries displayed a strong PKC-βII signal in DM rats (figure 5C). In contrast, the TRITC signals of anti-PKC-βII antibodies were weak in control and DM+cur rat vessels (figure 5B and 5D, respectively). Negative controls displayed minimal detectable fluorescence when the secondary antibodies were used alone (figure 5A).

### Correlation between intracellular superoxide production and arteriolar vasodilation

**Figure 6:**
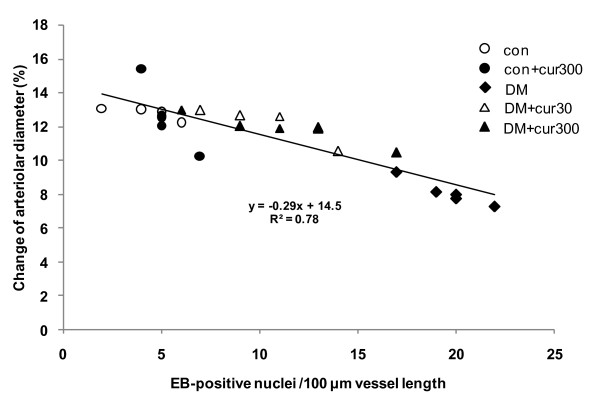
**Relationship between ethidium bromide-positive nuclei and % changes in ACh-induced arteriolar vasodilation**. Relationship between EB-positive nuclei per 100 μm vessel length and percentage of ACh-induced change in arteriolar diameter for diabetes (DM), diabetes treated with 30 and 300 mg/kg curcumin (DM+cur30 and DM+cur300, respectively), control (con) and control treated with 300 mg/kg curcumin (con+cur300).

To examine the correlation between intracellular superoxide production and endothelial vascular response, figure 6 shows the relationship between superoxide production and ACh-induced arteriolar vasodilation, for every group. The results indicated that the EB-positive nuclei along the mesenteric arterioles and the percent changes in arteriolar diameters stimulated by ACh have a significant correlation (0.78, *P *< 0.01). This correlation is described by the linear equation:

$$\text{y}=-0.\text{29x}+\text{14}.\text{5 (}R^{2}=0.\text{78},P<0.0\text{1)}$$

Where x is the number of EB-positive nuclei per 100 um vessel length and y is the percentage change in arteriolar diameter.

## Discussion

In the present study, we have shown that the effect of curcumin supplementation on diabetes-induced endothelial dysfunction is closely associated with its potential as an anti-oxidant. The supplementation of either low or high doses of curcumin appears to improve diabetic endothelial dysfunction, as shown by the increase in ACh-activated vasodilation. However, there was no significant difference between low and high doses in terms of restoring effects. In contrast to this increase in ACh-vasorelaxation, endothelium-independent relaxation in response to the NO donor SNP was not affected by either diabetes or curcumin supplementation. Therefore in this twelve-week model of diabetes, the NO-stimulated cGMP signaling in arteriolar smooth muscle was not the primary target of treatment.

It is well established that hyperglycemia can produce ROS production by a series of cellular events and further leads to diabetic complications due to oxidative stress [2,21]. Previously, a diabetes-induced increase in ROS was indirectly demonstrated using lipid peroxidation end-products (e.g., malondialdehyde [MDA]) as an indicator [10,17].

In order to examine the dynamic process of diabetes-stimulated ROS production and its correlation with endothelial dysfunction, our study utilized hydroethidine-sensitive vascular superoxide detection. One of the most frequently used assays for the detection of cellular O_2_^●- ^production utilizes hydroethidine as an intracellular probe [22,23]. In the presence of O_2_^●-^, hydroethidine is rapidly converted to ethidium bromide, which binds to DNA and is detected by its red fluorescent light following minimal oxidation induced by H_2_O_2_, ONOO^-^, or HOCl^- ^[24]. *In situ *nuclei labeled with ethidium bromide along the arteriolar wall could be observed and the number of EB-sensitive nuclei could be quantitatively estimated per 100 microns of vessel length, as shown in Figure 4.

The results showed that superoxide production along the arteriolar wall was about 4.45 times higher in DM rats than controls. This excessive increase in vascular superoxide may have destroyed the vascular endothelial lining, yielding a 0.67-fold decrease in Ach response.

Interestingly, our findings have indicated that this 4.45-fold increase in superoxide production in diabetic vasculature could be attenuated by daily oral curcumin supplementation. However, there was no difference between the low and high doses of curcumin in terms of reducing superoxide production at the diabetic vascular wall. Both doses of curcumin examined in this study were able to decrease superoxide production by almost two-fold. Curcumin has been reported as a potent scavenger of a variety of ROS [25], exhibiting anti-inflammatory activity as well as antioxidant properties [17,26-29]. The phenolic (OH) structure of curcumin was believed to be essential for curcumin's anti-oxidant activity [28].

In addition, the anti-oxidant effect of curcumin on protecting endothelial function against ROS damage may be partially attributed to the hypoglycemic effect of curcumin. Our findings are consistent with others showing that treatment with curcumin in diabetic rats leads to lower plasma glucose levels [17,29]. However, the new finding in the present study was that the hypoglycemic potential of curcumin is dose dependent (Table 1). Since HbA1c is the product of non-enzymatic glycosylation, therefore, it is mostly a better indicator of glycemic control than plasma glucose level. Although the mechanisms underlying the anti-diabetic action of curcumin remain unknown, it has been suggested by other investigators that curcumin may inhibit hepatic glucose output and/or stimulate insulin secretion from the pancreas [26,30,31]. Moreover, it has been reported recently that the anti-diabetic potential of anti-oxidants such as vitamin C could protect glucose transporter 1 (GLUT-1) [32]. Therefore, the hypoglycemic effect of curcumin may be attributed to this effect on GLUT-1 as well.

STZ induces cytotoxicity mediated by reactive oxygen species, as evidenced by B cell damage. Therefore, it is possible that the hypoglycemic effect of curcumin, mediated by stimulating insulin secretion from the pancreas, may be limited by the number of B cells remaining. Therefore, the antidiabetic effect of curcumin should be further clarified with the understanding that curcumin supplementation cannot be used as an antidiabetic on its own.

The increase in hyperglycemia-induced oxygen-derived free radicals was believed to be a major contributor to the reduction of NO bioavailability observed in diabetes. The interaction between NO and O_2_^●- ^occurs at an extremely rapid rate, three times faster than the rate of O_2_^●- ^reaction with SOD [33]. Therefore, this hyperglycemia-induced O_2_^●- ^may quench NO, preventing endothelium-dependent vasodilation [34-36].

Our findings showed that both low and high doses of curcumin could significantly lower blood glucose by 18.73% and 30.26%, respectively, in the diabetic group. In addition, the results also showed that both low and high doses of curcumin could decrease diabetic vascular superoxide production down by 55.1% and 57.1%, respectively. Simultaneously, both doses of curcumin were able to increase ACh-activated vasodilatation by up to 30.22% and 46.47%, respectively. Although our study did not monitor NO production directly, this parameter can be measured indirectly by quantifying endothelial-dependent vasodilation. Our findings in this regard indicate that curcumin supplementation could enhance endothelial-dependent relaxation in diabetic rats. Johnson et al. used *in vitro *studies to determine that the mechanism of curcumin-mediated protection against NO oxidation involves the sequestration of reaction intermediates [35]. The IC_50 _for curcumin with 1.0 μM DEA/NO was calculated to be 13 μM. Moreover, the authors also suggested that the mechanism of curcumin action involved the sequestration of NO_2 _but not NO.

Previous studies in rat aortic rings also demonstrated that curcumin (10(-11) mol/L) could alleviate the acute increase in glucose levels induced by dysfunctional endothelium-dependent vasodilation [37]. The authors suggested that the effect of curcumin may be due to its ability to enhance heme oxygenase and guanylate cyclase (GC) activity. Ach as well as NO-stimulated cGMP signaling is required for normal endothelium-dependent vasodilation. Therefore, the protective effect of curcumin on vasodilatation could be completely eliminated by the non-selective guanylate cyclase (GC) inhibitor methylene blue [37].

A clinical study by Usharani *et al*. (2008) [38] showed that NCB-02 (two capsules containing curcumin 150 mg twice daily) significantly reduced the levels of malondialdehyde, ET-1, IL-6 and TNF[alpha] in type 2 diabetes patients. Therefore, the molecular mechanisms of curcumin-mediated increases in vascular NO bioavailability might be enhanced by its anti-oxidant properties and by its anti-inflammatory effects. Notably, the pharmacokinetics of curcumin have recently been found to be associated with many pathophysiologies via its actions on signaling networks such as the NF-κB and MAPK/ERK pathways [39,40].

Numerous studies have shown that both free fatty acids and high glucose levels in diabetes may activate PKC in various vascular cells via *de novo *synthesis of diacylglycerol (DAG) [41]. In addition, it has been reported that such activated PKC could facilitate increased O_2_^●- ^production through PKC-dependent activation of NAD(P)H oxidase in vascular cells [42,43]. Activated PKC result in sustained increases in the production of O_2_^●- ^and induce oxidative damage to diabetic blood vessels, and it also induces a number of pathogenic consequences by activating NF-κB and affecting the expression of endothelial nitric oxide synthetase (eNOS), endothelin-1(ET-1), vascular endothelial growth factor (VEGF), transforming growth factor-β (TGF-β) and plasminogen activator inhibitor-1 (PAI-1) [42,44]. Therefore, in the present study, the effect of high-dose curcumin on activated PKC was further investigated. Immunofluorescent micrographs revealed that diabetes-activated PKC expression was increased markedly in 12-week diabetic mesenteric arterial wall. Interestingly, the immunofluorescent micrograph indicated that curcumin supplementation, at a dose of 300 mg/kg, could suppress this diabetes-activated PKC expression (Figure 5D). This finding is in agreement with the previous report by Balasubramanyam *et al*. (2003), which determined that the dose-dependent ROS inhibitory effect of curcumin interfered mechanistically with PKC activity [45].

In order to confirm the importance of curcumin action, the correlation between HE-sensitive superoxide production and ACh-induced arteriolar vasodilation was examined for all five groups. These results were confirmed by the strong correlation between both parameters (0.78, *P *< 0.01).

## Conclusion

In conclusion, diabetes-induced endothelial dysfunction is closely associated with increases in oxidative stress along the vascular wall. Curcumin supplementation can improve diabetes-induced endothelial dysfunction through its ability to decrease O_2_^●-^production by inhibiting PKC. Curcumin supplementation may benefit diabetic patients by improving microvascular function and preventing cardiovascular complications.

## Competing interests

The authors declare that they have no competing interests.

## Authors' contributions

SR: Carried out the animal experiments, including Intra-vital set-up and immunoassays, and edited the first draft of the manuscript. ^1*^Contributed 45% of this work. NT: Participated in the experimental design and manuscript improvement. ^2*^Contributed 15% of this work. PR: Carried out sample selection and fixation. ^3*^Contributed 5% of this work. SP: Grant support and manuscript improvement, submission, and correspondence. ^4§^Contributed 35% of this work. All authors read and approved the final version of the manuscript.

## Pre-publication history

The pre-publication history for this paper can be accessed here:

http://www.biomedcentral.com/1472-6882/10/57/prepub

## References

[B1] UPDSU Group Intensive blood-glucose control with sulphonylureas or insulin compared with conventional treatment and risk of complications in patients with type 2 diabetes (UKPDS 33) Lancet 1998 352 9131 837 53 10.1016/S0140-6736(98)07019-6 9742976

[B2] Guzik TJ Mussa S Gastaldi D Sadowski J Ratnatunga C Pillai R Mechanisms of increased vascular superoxide production in human diabetes mellitus: role of NAD(P)H oxidase and endothelial nitric oxide synthase Circulation 2002 105 14 1656 62 10.1161/01.CIR.0000012748.58444.08 11940543

[B3] Droge W Free radicals in the physiological control of cell function Physiol Rev 2002 82 1 47 95 11773609 10.1152/physrev.00018.2001

[B4] Hink U Li H Mollnau H Oelze M Matheis E Hartmann M Mechanisms underlying endothelial dysfunction in diabetes mellitus Circ Res 2001 88 2 E14 22 11157681 10.1161/01.res.88.2.e14

[B5] Katusic ZS Superoxide anion and endothelial regulation of arterial tone Free Radic Biol Med 1996 20 3 443 8 10.1016/0891-5849(96)02116-8 8720916

[B6] Rubanyi GM Vanhoutte PM Superoxide anions and hyperoxia inactivate endothelium-derived relaxing factor Am J Physiol 1986 250 5 Pt 2 H822 7 3010744 10.1152/ajpheart.1986.250.5.H822

[B7] Lund DD Faraci FM Miller FJ Jr Heistad DD Gene transfer of endothelial nitric oxide synthase improves relaxation of carotid arteries from diabetic rabbits Circulation 2000 101 9 1027 33 10704171 10.1161/01.cir.101.9.1027

[B8] Mugge A Elwell JH Peterson TE Hofmeyer TG Heistad DD Harrison DG Chronic treatment with polyethylene-glycolated superoxide dismutase partially restores endothelium-dependent vascular relaxations in cholesterol-fed rabbits Circ Res 1991 69 5 1293 300 1934359 10.1161/01.res.69.5.1293

[B9] Wohaieb SA Godin DV Alterations in free radical tissue-defense mechanisms in streptozocin-induced diabetes in rat. Effects of insulin treatment Diabetes 1987 36 9 1014 8 10.2337/diabetes.36.9.1014 3301471

[B10] Jariyapongskul A Patumraj S Yamaguchi S Niimi H The effect of long-term supplementation of vitamin C on leukocyte adhesion to the cerebral endothelium in STZ-induced diabetic rats Clin Hemorheol Microcirc 2002 27 1 67 76 12237491

[B11] Sridulyakul P Chakraphan D Patumraj S Vitamin C supplementation could reverse diabetes-induced endothelial cell dysfunction in mesenteric microcirculation in STZ-rats Clin Hemorheol Microcirc 2006 34 1-2 315 21 16543652

[B12] Timimi FK Ting HH Haley EA Roddy MA Ganz P Creager MA Vitamin C improves endothelium-dependent vasodilation in patients with insulin-dependent diabetes mellitus J Am Coll Cardiol 1998 31 3 552 7 10.1016/S0735-1097(97)00536-6 9502634

[B13] Taniyama Y Griendling KK Reactive oxygen species in the vasculature: molecular and cellular mechanisms Hypertension 2003 42 6 1075 81 10.1161/01.HYP.0000100443.09293.4F 14581295

[B14] Bengmark S Curcumin, an atoxic antioxidant and natural NFkappaB, cyclooxygenase-2, lipooxygenase, and inducible nitric oxide synthase inhibitor: a shield against acute and chronic diseases JPEN J Parenter Enteral Nutr 2006 30 1 45 51 10.1177/014860710603000145 16387899

[B15] Masuda T Maekawa T Hidaka K Bando H Takeda Y Yamaguchi H Chemical studies on antioxidant mechanism of curcumin: analysis of oxidative coupling products from curcumin and linoleate J Agric Food Chem 2001 49 5 2539 47 10.1021/jf001442x 11368633

[B16] Masuda T Toi Y Bando H Maekawa T Takeda Y Yamaguchi H Structural identification of new curcumin dimers and their contribution to the antioxidant mechanism of curcumin J Agric Food Chem 2002 50 9 2524 30 10.1021/jf011601s 11958616

[B17] Patumraj S Wongeakin N Sridulyakul P Jariyapongskul A Futrakul N Bunnag S Combined effects of curcumin and vitamin C to protect endothelial dysfunction in the iris tissue of STZ-induced diabetic rats Clin Hemorheol Microcirc 2006 35 4 481 9 17148847

[B18] Thomas G Roques B Proton magnetic resonance studies of ethidium bromide and its sodium borohydride reduce derivative FEBS Letters 1972 26 169 75 10.1016/0014-5793(72)80566-0

[B19] Suzuki H DeLano FA Parks DA Jamshidi N Granger DN Ishii H Xanthine oxidase activity associated with arterial blood pressure in spontaneously hypertensive rats Proc Natl Acad Sci USA 1998 95 8 4754 9 10.1073/pnas.95.8.4754 9539811 PMC22563

[B20] Suzuki H Swei A Zweifach BW Schmid-Schonbein GW In vivo evidence for microvascular oxidative stress in spontaneously hypertensive rats. Hydroethidine microfluorography Hypertension 1995 25 5 1083 9 7737720 10.1161/01.hyp.25.5.1083

[B21] Brownlee M Biochemistry and molecular cell biology of diabetic complications Nature 2001 414 6865 813 20 10.1038/414813a 11742414

[B22] Bizyukin A Soodaeva S Study of oxidative phagocyte metabolism using the fluorescent indicator hydroethidine Pharmaceu Chem J 1995 29 236 40 10.1007/BF02219542

[B23] Carter WO Narayanan PK Robinson JP Intracellular hydrogen peroxide and superoxide anion detection in endothelial cells J Leukoc Biol 1994 55 2 253 8 8301222 10.1002/jlb.55.2.253

[B24] Benov L Sztejnberg L Fridovich I Critical evaluation of the use of hydroethidine as a measure of superoxide anion radical Free Radic Biol Med 1998 25 7 826 31 10.1016/S0891-5849(98)00163-4 9823548

[B25] Reddy AC Lokesh BR Studies on the inhibitory effects of curcumin and eugenol on the formation of reactive oxygen species and the oxidation of ferrous iron Mol Cell Biochem 1994 137 1 1 8 10.1007/BF00926033 7845373

[B26] Halim E Hussain M Hypoglycemic, hypolipidemic and antioxidant properties of combination of Curcumin from *CURCUMA LONGA*, Linn, and partially purified product from ABROMA AUGUSTA, Linn, in streptozotocin induced diabetes Indian J Clin Biochem 2002 17 33 43 10.1007/BF02867969 PMC3454113 23105348

[B27] Kunchandy E Rao M Oxygen radical scavenging activity of curcumin Int J Pharm 1990 58 3 237 40 10.1016/0378-5173(90)90201-E

[B28] Priyadarsini KI Maity DK Naik GH Kumar MS Unnikrishnan MK Satav JG Role of phenolic O-H and methylene hydrogen on the free radical reactions and antioxidant activity of curcumin Free Radic Biol Med 2003 35 5 475 84 10.1016/S0891-5849(03)00325-3 12927597

[B29] Sharma S Kulkarni SK Chopra K Curcumin, the active principle of turmeric (Curcuma longa), ameliorates diabetic nephropathy in rats Clin Exp Pharmacol Physiol 2006 33 10 940 5 10.1111/j.1440-1681.2006.04468.x 17002671

[B30] Lochhead PA Salt IP Walker KS Hardie DG Sutherland C 5-aminoimidazole-4-carboxamide riboside mimics the effects of insulin on the expression of the 2 key gluconeogenic genes PEPCK and glucose-6-phosphatase Diabetes 2000 49 6 896 903 10.2337/diabetes.49.6.896 10866040

[B31] Yamauchi T Kamon J Minokoshi Y Ito Y Waki H Uchida S Adiponectin stimulates glucose utilization and fatty-acid oxidation by activating AMP-activated protein kinase Nat Med 2002 8 11 1288 95 10.1038/nm788 12368907

[B32] Kim DI Lim SK Park MJ Han HJ Kim GY Park SH The involvement of phosphatidylinositol 3-kinase/Akt signaling in high glucose-induced downregulation of GLUT-1 expression in ARPE cells Life Sci 2007 80 7 626 32 10.1016/j.lfs.2006.10.026 17141276

[B33] Benz D Cadet P Mantione K Zhu W Stefano G Tonal nitric oxide and health--a free radical and a scavenger of free radicals Med Sci Monit 2002 8 1 RA1 4 11782688

[B34] Jariyapongskul A Patumraj S Niimi H Cerebral endothelial dysfunction in diabetes: intravital microscopic analysis using streptozotocin-induced diabetic rats Clin Hemorheol Microcirc 2003 29 3-4 331 5 14724358

[B35] Johnston BD DeMaster EG Suppression of nitric oxide oxidation to nitrite by curcumin is due to the sequestration of the reaction intermediate nitrogen dioxide, not nitric oxide Nitric Oxide 2003 8 4 231 4 10.1016/S1089-8603(03)00030-2 12895432

[B36] Meininger CJ Marinos RS Hatakeyama K Martinez-Zaguilan R Rojas JD Kelly KA Impaired nitric oxide production in coronary endothelial cells of the spontaneously diabetic BB rat is due to tetrahydrobiopterin deficiency Biochem J 2000 349 Pt 1 353 6 10.1042/0264-6021:3490353 10861247 PMC1221156

[B37] Fang XD Yang F Zhu L Shen YL Wang LL Chen YY Curcumin ameliorates high glucose-induced acute vascular endothelial dysfunction in rat thoracic aorta Clin Exp Pharmacol Physiol 2009 36 12 1177 82 10.1111/j.1440-1681.2009.05210.x 19473193

[B38] Usharani P Mateen A Naidu M Raju Y Chandra N Effect of NCB-02, Atorvastatin and placebo on endothelial function, oxidative stress and inflammatory markers in patients with T2 diabetes mellitus: A randomized, parallel-group, placebo-controlled, 8-week study Drug in R&D 2008 9 4 243 50 10.2165/00126839-200809040-00004 18588355

[B39] Aggarwal BB Sung B Pharmacological basis for the role of curcumin in chronic diseases: an age-old spice with modern targets Trends Pharmacol Sci 2009 30 2 85 94 10.1016/j.tips.2008.11.002 19110321

[B40] Patumraj S Yoysungneon P Curcumin as a therapeutic agent against cancer Asian Biomedicine 2007 1 239 52

[B41] Beckman JA Goldfine AB Gordon MB Garrett LA Creager MA Inhibition of protein kinase Cbeta prevents impaired endothelium-dependent vasodilation caused by hyperglycemia in humans Circ Res 2002 90 1 107 11 10.1161/hh0102.102359 11786526

[B42] Inoguchi T Li P Umeda F Yu HY Kakimoto M Imamura M High glucose level and free fatty acid stimulate reactive oxygen species production through protein kinase C--dependent activation of NAD(P)H oxidase in cultured vascular cells Diabetes 2000 49 11 1939 45 10.2337/diabetes.49.11.1939 11078463

[B43] Pacher P Beckman JS Liaudet L Nitric oxide and peroxynitrite in health and disease Physiol Rev 2007 87 1 315 424 10.1152/physrev.00029.2006 17237348 PMC2248324

[B44] Wolin MS Gupte SA Oeckler RA Superoxide in the vascular system J Vasc Res 2002 39 3 191 207 10.1159/000063685 12097818

[B45] Balasubramanyam M Adaikala Koteswari A Sampath Kumar R Finny Monickaraj S Uma Maheswari J Mohan V Curcumin-induced inhibition of cellular reactive oxygen species generation: Novel therapeutic implications J Biosciences 2003 28 6 715 21 10.1007/BF02708432 14660871

